# Temperature Drift Compensation of a MEMS Accelerometer Based on DLSTM and ISSA

**DOI:** 10.3390/s23041809

**Published:** 2023-02-06

**Authors:** Gangqiang Guo, Bo Chai, Ruichu Cheng, Yunshuang Wang

**Affiliations:** Xi’an Microelectronics Technology Institute, Xi’an 710054, China

**Keywords:** MEMS accelerometer, temperature drift, real-time compensation model, DLSTM + ISSA

## Abstract

In order to improve the performance of a micro-electro-mechanical system (MEMS) accelerometer, three algorithms for compensating its temperature drift are proposed in this paper, including deep long short-term memory recurrent neural network (DLSTM-RNN, short DLSTM), DLSTM based on sparrow search algorithm (SSA), and DLSTM based on improved SSA (ISSA). Moreover, the piecewise linear approximation (PLA) method is employed in this paper as a comparison to evaluate the impact of the proposed algorithm. First, a temperature experiment is performed to obtain the MEMS accelerometer’s temperature drift output (TDO). Then, we propose a real-time compensation model and a linear approximation model for neural network methods compensation and PLA method compensation, respectively. The real-time compensation model is a recursive method based on the TDO at the last moment. The linear approximation model considers the MEMS accelerometer’s temperature and TDO as input and output, respectively. Next, the TDO is analyzed and optimized by the real-time compensation model and the three algorithms mentioned before. Moreover, the TDO is also compensated by the linear approximation model and PLA method as a comparison. The compensation results show that the three neural network methods and the PLA method effectively compensate for the temperature drift of the MEMS accelerometer, and the DLSTM + ISSA method achieves the best compensation effect. After compensation by DLSTM + ISSA, the three Allen variance coefficients of the MEMS accelerometer that bias instability, rate random walk, and rate ramp are improved from $5.43\times 10^{-4}\;\mathrm{mg}$, $4.33\times 10^{-5}\;{\mathrm{mg}/s}^{\frac{1}{2}}$, $1.18\times 10^{-6}\;\mathrm{mg}/s$ to $2.77\times 10^{-5}\;\mathrm{mg}$, $1.14\times 10^{-6}\;{\mathrm{mg}/s}^{\frac{1}{2}}$, $2.63\times 10^{-8}\;\mathrm{mg}/s$, respectively, with an increase of 96.68% on average.

## 1. Introduction

The MEMS accelerometer is one of the essential measurement elements of an Inertial Measurement Unit (IMU), which inherits the advantages of MEMS technology, such as small size, light weight, low cost, low power consumption, etc. MEMS accelerometers can be used in various fields, such as aerospace, healthcare, gait analysis, sport science, activity recognition, and portable devices [1,2,3,4,5,6,7,8,9]. However, existing manufacturing defects, such as manufacturing tolerances, always degrade its performance. In addition, MEMS accelerometers are extremely sensitive to ambient temperature, causing their performance to degrade dramatically with temperature, which limits their application in high-precision fields. In recent years, researchers have proposed many different approaches to address the effects of temperature on MEMS accelerometers. In general, they are mainly divided into two methods: hardware method and software method.

The hardware method improves the temperature characteristics of MEMS accelerometers mainly through circuit control and structural optimization, which always require additional time and economic costs. Wang proposed a temperature compensation method for a high-performance resonant MEMS accelerometer based on a control circuit and structural design [10]. Liu used parasitic resistance to compensate for the temperature drift of high precision capacitive accelerometers [11]. Tsai optimized the temperature drift of the accelerometer by limiting the distribution area of different materials to suppress the mismatch of expansion coefficients [12]. Zotov used two identical tuning forks with opposite sensitive axes to eliminate common mode errors to suppress temperature effects [13]. Jing collected the temperature inside the accelerometer package through an on-chip temperature sensor to more accurately compensate for temperature effects [14]. Kose eliminated thermal lag between the DETF resonator and accelerometer for more accurate temperature, enabling more accurate temperature compensation of capacitive MEMS accelerometers [15]. Ma created a common-mode signal based on a modulated feedback architecture and added the common-mode signal to the closed loop to compensate for the temperature drift of the MEMS capacitive accelerometer [16]. Zhang introduced an excitation signal to drive the proof mass and demodulated the response amplified by the mechanical stiffness and readout gain to compensate for the temperature drift of the MEMS accelerometer [17]. Parmar implemented temperature compensation of MEMS capacitive accelerometers with suitable TC circuits [18].

The software method usually studies the mathematical relationship between the temperature and the temperature drift output through modeling and then calculates the model parameters through mathematical methods to achieve compensation. This approach is often time-saving, money-saving, and easy to implement. He studied the relationship between thermal deformation and stiffness temperature dependence and presented an analytical study and a compensation structure for temperature drifts of a bulk silicon MEMS capacitive accelerometer. After compensation, the bulk silicon MEMS capacitive accelerometer’s temperature drift was suppressed by 71.89% [19]. Ruzza used low-order polynomials to compensate for the thermal effects of low-cost MEMS accelerometers to solve the common drawbacks that currently make low-cost MEMS sensors unsuitable for tilt-based monitoring applications. The author developed a miniaturized temperature-controlled oven and mounted it on a tilting device to account for tilt angle variation. The results show that this paper’s low-cost MEMS accelerometer’s RMS errors decreased by 96% [20]. Zhang adopted the method of finite element analysis to identify the parameters of the temperature drift model to realize the compensation of the capacitive MEMS accelerometer [21]. Khankalantary studied the relationship between the error coefficients and temperature and employed cubic spline interpolation to model the relationship to remove temperature effects [22]. Wang eliminated the temperature effects of MEMS resonant accelerometers with an optimized back propagation neural network (BP NN) [23]. Han aimed to improve the accuracy of MEMS capacitive accelerometers over temperature through a BP NN model and an adaptive genetic algorithm (AGA) in rapidly changing temperature environments. The traditional genetic algorithm (GA) can realize an extensive search for optimal solutions in the solution space but easily falls into local minima because of the constant probabilities of crossover and mutation. The characteristic of the proposed AGA is nonlinearly adjusting the probabilities. The validation results show that AGA-BP has the best compensation effect [24]. Qi simulated a MEMS accelerometer’s structural deformation in diverse conditions to trace its temperature drift error (TDE). Then, the author considered the ambient temperature, ambient temperature change, and the square of the two in the temperature drift model and improved the accuracy of the MEMS accelerometer with temperature changes through the BP NN model based on particle swarm optimization (PSO) and GA. GA is used to remove local optimums of BP NN, and PSO is utilized to solve the probabilistic disorder of GA to improve its accuracy [25]. Zhu focused on compensating the temperature drift of high-G MEMS accelerometers through a fusion algorithm. In this paper, the author considered temperature, temperature variation rate, and temperature product terms to model temperature drift. Then, the radial basis function neural networks (RBF NN) optimized by a Kalman filter (KF) and GA was proposed, and the temperature model’s output corrected its output. At this time, the fusion algorithm is determined [26]. Du studied the real-time temperature compensation algorithm of a force-rebalanced MEMS capacitive accelerometer based on the linear relationship between its temperature and dynamic resonant frequency. After compensation, the first-order temperature coefficient of the accelerometer’s bias offset improved by 98.59%, and the long-term drift was suppressed by 93.14% [27]. Han analyzed the temperature effect and proposed the temperature compensation of a MOEMS accelerometer. The author used a finite-element method to complete the quantitative analysis and set up the temperature model by simulating the deformation of the sensor chip. The model was used to describe the accelerometer’s temperature characteristics, and the temperature compensation was put forward based on the model. The results show that the temperature compensation can improve the stability of the MOEMS accelerometer [28]. Considering some specific applications, Yang considered a simple mathematical model to compensate for low-cost quartz accelerometers’ temperature drifts at high temperatures. His model considers both temperature and rolling sensitivity and has been successfully applied to the temperature compensation of two low-cost quartz accelerometers. The result shows that the temperature drift was suppressed by 90% [29]. Pan focused on the compensation of bias drift and scale factor for a quartz flexible accelerometer between a quick turn-on and the thermal balance inside the system based on a three-layer depth wavelet neural network. Moreover, a variable rate learning algorithm was also employed in this method. Results show that the compensation algorithm increases efficiency and improves precision [30]. Li proposed an improved BP NN based on GA for temperature drift compensation of a quartz flexible accelerometer in different temperatures. The accelerometer’s temperature and temperature drift output are used as the input and output of the model, respectively. The GA operation(selection, crossover, and mutation) improves the BP NN’s optimization capability. The results show that this method achieved a good effect and had fewer training steps and better fitting accuracy precision than the standard BP NN [31]. Yu employed the artificial fish swarm (AFS) algorithm to carry out temperature drift compensation for a quartz flexible accelerometer. The accelerometer’s temperature and output voltages are the input and output of the compensation model. The results show that the accelerometer’s drift instability is reduced by 88.76% after compensation by the AFS algorithm [32]. Wu studied a quartz flexible accelerometer’s temperature characteristics and proposed a method for its temperature drift compensation in the cold start condition. The author established the temperature drift compensation models based on temperature, temperature gradient, and time-related drift. Then, the PSO algorithm was employed to find the best model parameters. The results show that the proposed method greatly reduces the output signal’s standard deviation [33].

Unlike the previous studies, this paper proposes a real-time compensation model and introduces a deep-learning approach. Moreover, a swarm intelligence optimization, SSA, is used to tune the parameters of DLSTM for finding the optimal global parameters, and an improved method is proposed to enhance the optimization ability of SSA. For evaluating the proposed methods’ influence, the linear approximation model and the PLA method are introduced in this paper as a comparison.

This paper proposes three methods for temperature drift compensation of a MEMS accelerometer: DLSTM, DLSTM + SSA, and DLSTM + ISSA. First, the experimental product, named guidance navigation and control (GNC) module, and the temperature experimental process are briefly introduced. Second, a real-time model is described to study the characteristics of the temperature drift of the MEMS accelerometer, which is a recursive method whose input and output are the output of the temperature drift at the last moment and the current moment, respectively. We then aim to improve the accuracy of the previously proposed model with DLSTM, a kind of RNN variant, since the temperature drift of MEMS accelerometers is a random time series, and RNNs are good at this signal. Afterwards, the parameters of DLSTM are optimized by SSA and ISSA, respectively. More accurate compensation results are obtained, further improving the generalization ability and robustness of the real-time model and DLSTM. Moreover, the linear approximation model and PLA method are also employed as a comparison with the proposed methods in this paper. Finally, Allan variance analysis is used to quantify the compensation results of the four methods. The results verify that the DLSTM + ISSA method shows the best compensation performance.

## 2. Temperature Experiment

### 2.1. Experimental Product

**Figure 1:**
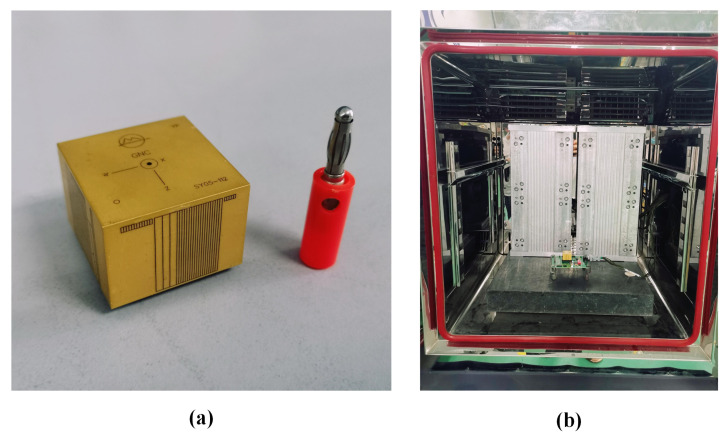
(**a**) The In-house-designed GNC Module; (**b**) temperature test equipment.

The experimental product used in this paper is an in-house-designed GNC Module developed entirely by us. The GNC Module includes a dual-core processor, AD, IMU, external interface, etc., which can provide a full range of inertial information. In addition, combined with information from other external sensors, such as GPS or geomagnetic field information, the GNC Module can realize a broader range of functions, such as integrated navigation, flight control, electronic control combination, steering gear control, etc. The dimensions of the GNC Module are 30 mm × 30 mm × 21.6 mm. Its picture is shown in Figure 1a.

### 2.2. Experimental Scheme

In order to study the temperature drift characteristics of the MEMS accelerometer in the GNC Module, we conducted a temperature test on it. Here, we ignore the influence of other factors and set the reference value of temperature drift equal to zero. The experimental scheme is set up as follows.

1. As is shown in Figure 1b, the GNC Module is placed in a temperature-controlled oven with a static base The accelerometer chosen in this paper has axes perpendicular to the local gravity direction. The function of the static base is to ensure that the product on it is not disturbed by the vibration of the external environment;
2. At the beginning of the experiment, the temperature of the temperature-controlled oven is set to −40 °C and maintained for 1 h;
3. The temperature of the temperature-controlled oven is raised to 80 °C at a heating rate of 1 °C/min. At the beginning of this step, a computer outside the temperature-controlled oven synchronously collected the MEMS accelerometer’s output with a frequency of 200 Hz until the end of the heating process;
4. The temperature of the temperature-controlled oven is maintained at 80 °C for 1 h;
5. Then the temperature of the temperature-controlled oven is brought down to room temperature. The temperature experiment is over.

## 3. Model and Algorithm

### 3.1. Neural Network Methods

#### 3.1.1. Real-Time Compensatiom Model

**Figure 2:**
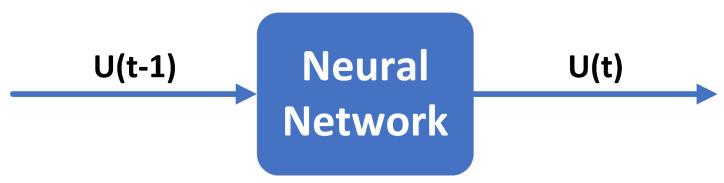
The real-time compensatiom model.

Many authors typically use temperature and other temperature-related quantities as inputs to temperature compensation models. In this way, the collected temperature must be accurate enough. Otherwise, the temperature error will be directly introduced into the compensation model, thus reducing the compensation accuracy. In addition, the temperature is usually output synchronously with temperature drift, so real-time compensation is impossible. We propose a one-step ahead predictor to perform real-time compensation, a recursive method based on the TDO from the last moment. This method uses the TDO at time t−1 (U(t−1)) and *t* (U(t)) as the model input and output, respectively. That is, the TDO of the MEMS accelerometer from 1 to M−1 ([U(1),U(2),…,U(M−1)]) and from 2 to *M* ([U(2),U(3),…,U(M)]) are represented as the input and output data for the proposed model, respectively. *M* is the total number of the MEMS accelerometer’s TDO. The model can be described as follows:

(1) Y(t)=U(t)=F[X(t)]=F[U(t−1)]=F[Y(t−1)]

where X(t) and Y(t) are the input and output of the model at time *t*, respectively. U(t−1) and U(t) are the TDO of the MEMS accelerometer at time t−1 and *t*, respectively. F[·] is the target function to be trained. The framework of the proposed real-time compensation model is shown in Figure 2.

#### 3.1.2. LSTM and DLSTM

**Figure 3:**
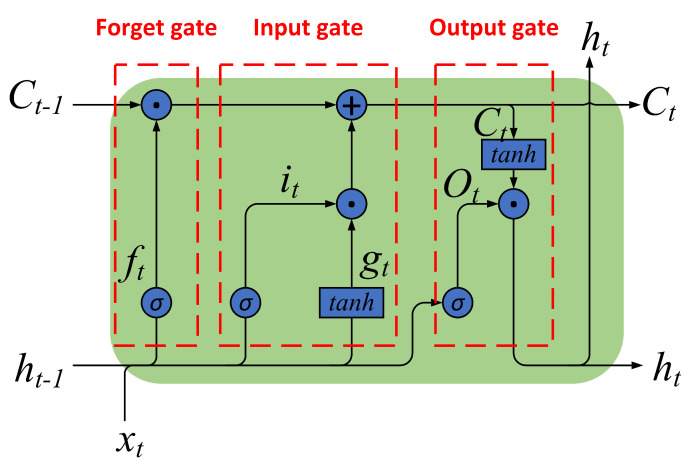
The framework of LSTM.

**Figure 4:**
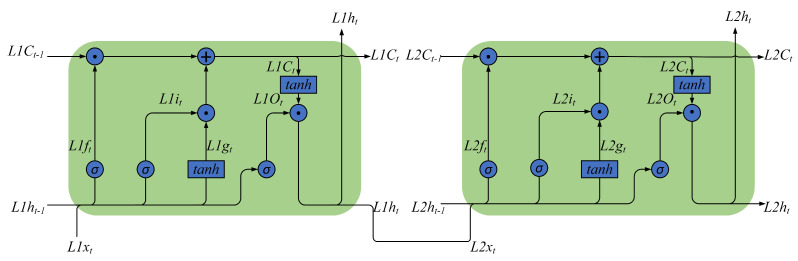
The framework of two-layer DLSTM.

**Figure 5:**
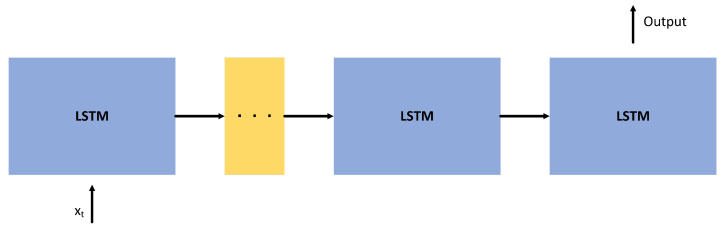
Basic structure of DLSTM workflow.

- LSTM
  LSTM was proposed by Hochreiter and Schmidhuber in 1997 and is a popular variant of RNN [34]. The framework of LSTM is shown in Figure 3.
  LSTM is built with a “gate” structure and a cell unit. Three gate units in LSTM adjust the information flow within the unit adaptively, called forget gate, input gate, and output gate. The cell can remember values over arbitrary time intervals [34,35,36]. The equations of the LSTM are described below.
  The sigmoid function implements the forget gate, which determines the information to be discarded from the cell state. The output of the forget gate ranges from zero to one, representing the forgetting degree of each input in the cell state. The smaller the forget gate output, the more information is forgotten. Its equation can be described as Formula (2).
  (2) $$f_{t}=\sigma \left(W_{fx}x_{t}+W_{fh}h_{t-1}+b_{f}\right)$$
  where $x_{t}$ and $h_{t-1}$ are the input of the forget gate, $W_{fx}$ and $W_{fh}$ are the coefficients of $x_{t}$ and $h_{t-1}$, respectively, $b_{f}$ is the threshold of forget gate, σ(·) is the sigmoid function, $f_{t}$ is the output of forget at time *t*, and $h_{t-1}$ is the output of LSTM at time *t*.
  The second part is the input gate, which determines the new information to be stored in the current cell state. The input gate consists of two parts. The first is the sigmoid function that decides the value to update. Similar to the forget gate, the output of the sigmoid function represents the updating degree of each input. The second is the tanh function that creates a new candidate value $g_{t}$. Both outputs are added to the cell state after a pointwise multiplication operation. Its equation can be described as Formulas (3) and (4).
  (3) $$i_{t}=\sigma \left(W_{ix}x_{t}+W_{ih}h_{t-1}+b_{i}\right)$$
  (4) $$g_{t}=\tan\;h\left(W_{gx}x_{t}+W_{gh}h_{t-1}+b_{g}\right)$$
  where tan h(·) is the tanh funtion. The meanings of other parameters are similar to the previous ones.
  The last part is the output gate, which decides what information to output. The function of the sigmoid function is similar to the previous which decides the output degree of each input. The cell state is put through a tanh function and then pointwise multiplication with $O_{t}$ as the output of LSTM. Its equation can be described as Formulas (5)–(7).
  (5) $$O_{t}=\sigma \left(W_{ox}x_{t}+W_{oh}h_{t-1}+b_{o}\right)$$
  (6) $$C_{t}=C_{t-1}⨀f_{t}+i_{t}⨀g_{t}$$
  (7) $$h_{t}=\tan\;h\left(C_{t}\right)⨀O_{t}$$
  where C(t) is the cell state at time *t*, and ⨀ represents the point-wise multiplication operation. The meanings of other parameters are similar to the previous ones.
  The above analysis shows that $x_{t}$ and $h_{t}$ are the input and output of LSTM, respectively; $f_{t}$, $i_{t}$, $g_{t}$, and O(t) are intermediate variables in the calculation process; $W_{fx}$, $W_{fh}$, $b_{f}$, $W_{ix}$, $W_{ih}$, $b_{i}$, $W_{gx}$, $W_{gh}$, $b_{g}$, $W_{ox}$, $W_{oh}$, and $b_{o}$ are the weights determined in the training process; σ(·) and tanh(·) are activation functions; C(t) records the cell state at time *t*, which is initialized as 0.
- DLSTM
  Figure 3 and Formulas (2)–(7) describe a single-layer LSTM. Figure 4 describes a two-layer DLSTM. In the two-layer DLSTM, the first-layer LSTM’s output is passed to the second-layer LSTM and used as input. The input vector goes through the two-layer DLSTM layer by layer to jointly determine the output of the two-layer DLSTM.
  In general, DLSTM is a sequence LSTM that decides the output together. Its structure is shown in Figure 5.

#### 3.1.3. SSA and ISSA

**Figure 6:**
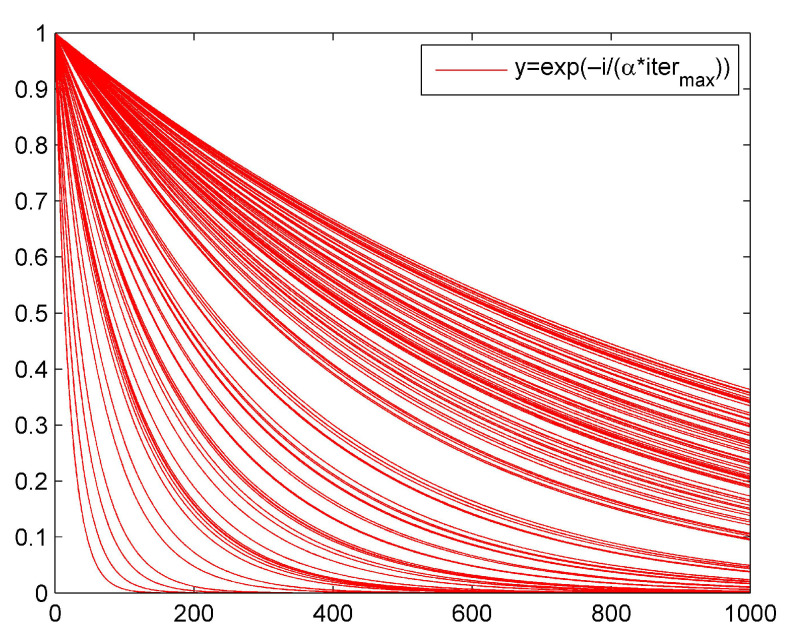
Distribution of $exp(-\frac{i}{\alpha \cdot iter_{max}})$.

**Figure 7:**
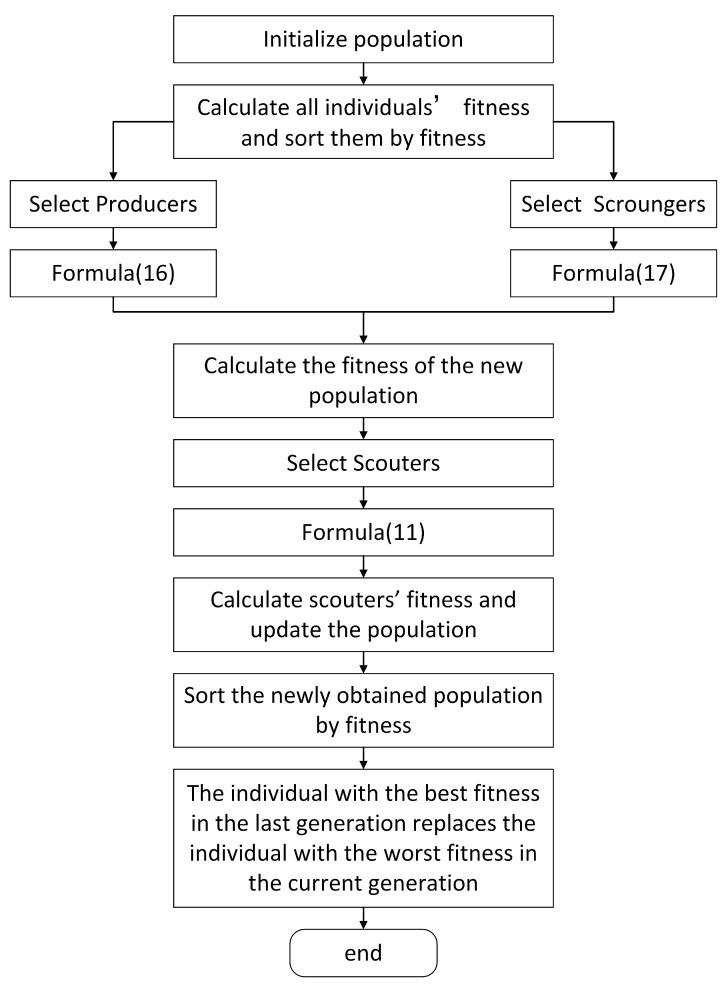
Workflow of ISSA.

- SSA
  SSA is a swarm intelligence optimization algorithm inspired by the foraging and anti-predation behaviors of sparrow populations, which Xue proposed in 2020 [37]. It has strong optimization capabilities and fast efficiency and was widely welcomed as soon as it was proposed. After simplifying and idealizing the biological characteristics of sparrow populations during foraging, the authors concluded the following rules.
  1. Sparrow populations typically divide during foraging into subpopulations called producers and scroungers. The criteria for classification are according to the fitness value of individual sparrows. Producers usually have better fitness values, while others are scroungers. Producers are primarily responsible for finding food and have larger search areas to provide foraging areas and directions for the entire sparrow population. Scroungers follow the producer’s location to find food;
  2. Some individuals in the population have a strong anti-predation ability, called scouters. When scouters detect danger, they will act against predation;
  3. Producers and scroungers can dynamically switch in each generation according to their fitness values, but the number of individuals in these two subpopulations remains the same;
  4. Some scroungers may monitor producers and compete for food, while some starving scroungers may look elsewhere for food;
  5. When aware of the danger, the fittest sparrow will randomly approach others, and others will move toward the best sparrow.
  Assume that there are a total of *N* sparrow individuals in the population, each with *d* dimensions/elements. This sparrow population can be represented as an expression (8). Each row of the matrix represents a sparrow individual.
  (8) $$\begin{array}{c} \left[\begin{array}{cccc} x_{1,1} & x_{1,2} & \ldots & x_{1,d}\\ x_{2,1} & x_{2,2} & \ldots & x_{2,d}\\ \vdots & \vdots & \ddots & \vdots \\ x_{N,1} & x_{N,2} & \ldots & x_{N,d} \end{array}\right] \end{array}$$
  The mathematical expression of SSA can be summarized as follows.
  **Updating Producer Location**
  (9) $$\begin{array}{c} x_{i,j}^{t+1}=\left\{\begin{array}{c} x_{i,j}^{t}\cdot exp\left(-\frac{i}{\alpha \cdot iter_{max}}\right),\;if\;R_{2}<ST\\ x_{i,j}^{t}+Q,\;if\;R_{2}\geq ST \end{array}\right. \end{array}$$
  where $x_{i,j}^{t}$ is the *j*-th dimension of the *i*-th individual in the *t*-th iteration of the producers; j=1,2,…,d, where *d* is the dimension of the sparrows; $iter_{max}$ is largest number of iterations; α is a random value in the range of (0,1]; *Q* is a random value from a normal distribution; $R_{2}\in \left[0,1\right]$ is the alarm value; ST∈[0.5,1] is the safety threshold; $R_{2}<ST$ means there is no danger around the producer, and they will continue their extensive search; and $R_{2}\geq ST$ means there is danger around the producer and they will fly to other safe areas.
  **Updating Scroungers Location**
  (10) $$\begin{array}{c} x_{i,j}^{t+1}=\left\{\begin{array}{c} Q\cdot exp\left(\frac{x_{worst,j}^{t}-x_{i,j}^{t}}{i^{2}}\right),\;if\;i>\frac{n}{2}\\ x_{best,j}^{t}+\left|x_{i,j}^{t}-x_{best,j}^{t}\right|\cdot A^{+}\cdot L,\;else \end{array}\right. \end{array}$$
  where $x_{best,j}^{t}$ and $x_{worst,j}^{t}$ are the *j*-th dimension of the individual with the best and worst fitness in the *t*-th iteration, respectively; *n* is the total number of sparrows; *A* is a *d* dimensional vector, each element of which is randomly 1 or −1, and $A^{+}=A^{T}{\left(AA^{T}\right)}^{-1}$; *L* is a *d* dimensional vector, each element of which is a 1; $i>\frac{n}{2}$ means that the fitness of *i*-th scrounger is poor, it will fly to other places for food; and $i\leq \frac{n}{2}$ means that the *i*-th scrounger is likely to forage around the best individual.
  **Updating Scouters Location**
  (11) $$\begin{array}{c} x_{i,j}^{t+1}=\left\{\begin{array}{c} x_{best,j}^{t}+\beta \cdot \left|x_{i,j}^{t}-x_{best,j}^{t}\right|,\;if\;f_{i}>f_{b}\\ x_{i,j}^{t}+K\cdot \left(\frac{|x_{i,j}^{t}-x_{worst,j}^{t}|}{(f_{i}-f_{w})+\epsilon }\right),\;if\;f_{i}=f_{b} \end{array}\right. \end{array}$$
  where β is a random number that follows a normal distribution; *K* is a random number that obeys a uniform distribution and K∈[−1,1]; ϵ is a very small constant to ensure the denominator is not 0; $f_{i}$,$f_{b}$, and $f_{w}$ are the *i*-th individual’s fitness, the best fitness, and the worst fitness in the *t*-th iteration, respectively; and $f_{i}=f_{b}$ and $f_{i}>f_{b}$ denote whether the sparrow is the individual with best fitness or not in the *t*-th iteration, respectively. When they feel danger, they follow Rule 5 to perform the corresponding actions, respectively.
- ISSA
  Although the original SSA has strong optimization ability and fast efficiency, it easily falls into the local optimum [38,39,40]. The foraging area and direction of the entire sparrow population mainly depend on the producers, so we need to expand the exploration range of the producers to improve the foraging ability of the entire population. The improvement of ISSA mainly comes from the following three aspects.
  1. Introducing self-adaptive hyper-parameters to producers: In Formula (9), the update position of the producer is affected by $exp(-\frac{i}{\alpha \cdot iter_{max}})$. When α is a random value, as *i* becomes larger, the value range of $exp(-\frac{i}{\alpha \cdot iter_{max}})$ becomes (0,0.4), where $iter_{max}=1000$. It is shown in Figure 6.
    We introduce the adaptive weight *w* shown by Formula (12) to improve the producer’s search speed and global search ability.
    (12) $$w=w_{0}\times c^{t}$$
    where $w_{0}=1$ is initial weight; *c* is set to 0.9.
    After adding the adaptive weight *w*, the producers’ formula is changed to Formula (13).
    (13) $$\begin{array}{c} X_{i,j}^{t+1}=\left\{\begin{array}{c} X_{i,j}^{t}\cdot exp\left(-\frac{i}{w\cdot \alpha \cdot iter_{max}}\right),\;if\;R_{2}<ST\\ X_{i,j}^{t}+Q,\;if\;R_{2}\geq ST \end{array}\right. \end{array}$$
  2. We Introduce the Lévy flight (LF) mechanism, which is widely used to overcome the premature convergence problem [41,42]. In the LF algorithm, the random walk is used to tune local search capabilities. The formula of LF is shown in Formulas (14) and (15).
    (14) $$LF=\frac{\sigma \cdot N_{LF}}{|M_{LF}{|}^{\frac{1}{\beta }}}$$
    (15) $$\sigma ={\left[\frac{\Gamma \left(1+\beta \right)\cdot sin\left(\frac{\pi \cdot \beta }{2}\right)}{\beta \Gamma \left(\frac{1+\beta }{2}\right)\cdot 2^{\frac{\beta -1}{2}}}\right]}^{\frac{1}{\beta }}$$
    where $M_{LF}$ and $N_{LF}$ are random numbers that obey a Gaussian distribution; β=1.5; and Γ(·) is Gamma function. After the introduction of the LF algorithm, Formulas (13) and (10) are changed to (16) and (17)
    (16) $$\begin{array}{c} X_{i,j}^{t+1}=\left\{\begin{array}{c} X_{i,j}^{t}\cdot exp\left(-\frac{i}{w\cdot \alpha \cdot iter_{max}}\right),\;if\;R_{2}<ST\\ X_{i,j}^{t}+LF,\;if\;R_{2}\geq ST \end{array}\right. \end{array}$$
    (17) $$\begin{array}{c} X_{i,j}^{t+1}=\left\{\begin{array}{c} LF\cdot exp\left(\frac{X_{worst,j}^{t}-X_{i,j}^{t}}{i^{2}}\right),\;if\;i>\frac{n}{2}\\ X_{best,j}^{t}+\left|X_{i,j}^{t}-X_{best,j}^{t}\right|\cdot A^{+}\cdot L,\;else \end{array}\right. \end{array}$$
  3. The sparrow individuals with the best fitness are directly passed on to the next generation. Its purpose is to ensure that the population’s overall quality does not decline.
  The workflow of ISSA is similar to SSA. The difference is that producers and scroungers obey different formulas in SSA and ISSA, respectively. Moreover, the individual with the best fitness in the last generation replaces the individual with the worst fitness in the current generation in ISSA. Here, we present the workflow by the example of ISSA. The workflow of ISSA is shown in Figure 7.

#### 3.1.4. Fusion Algorithm

**Figure 8:**
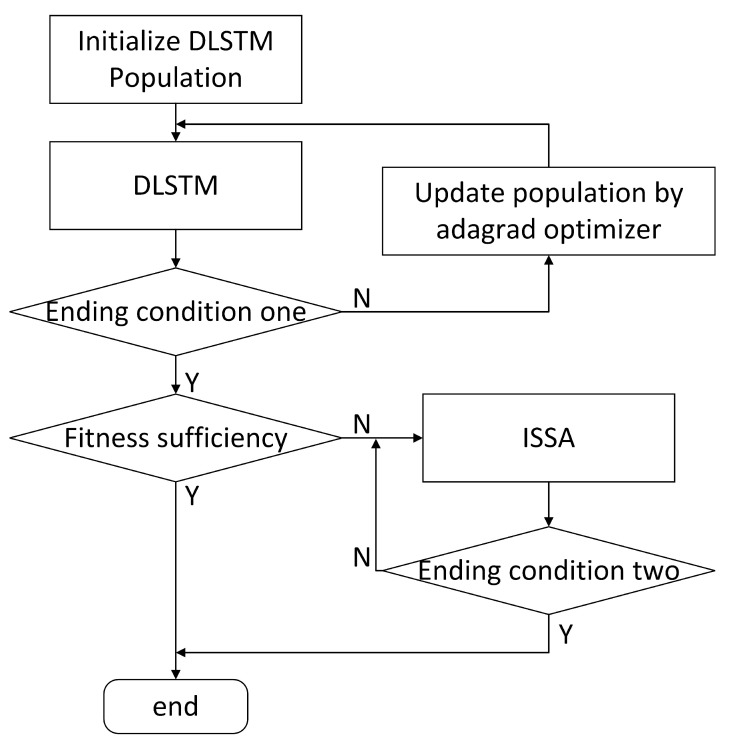
Workflow of DLSTM + ISSA.

It was introduced in the last section that ISSA is similar to SSA. In this section, we present the fusion algorithm by the example of DLSTM + ISSA. The fusion algorithm can improve the accuracy and generalization ability of the temperature drift model. The workflow of DLSTM + ISSA is shown in Figure 8, and its steps can be described as follows:

1. We randomly generate *N* groups values as the initial parameters of DLSTM. Each group of parameters represents a solution of DLSTM. Each group of parameters contains *d* elements, where *d* is the total number of parameters. These *N* groups of parameters have the same form as expression (8) and are used to train the DLSTM.
2. We train the DLSTM until the ending condition one is met; that is, the accuracy of the DLSTM meets requirements or reaches preset cycles of the DLSTM training. At this time, new *N* group parameters are obtained. If the accuracy meets the requirements, we finish training and export the optimal parameters. Otherwise, new *N* groups of parameters constitute the initial population of the ISSA.
3. The ISSA is used to evaluate and improve the parameters in the population further until ending condition two is met. That is, the accuracy of the DLSTM meets the requirements or reaches preset cycles of the ISSA.
4. We export the optimal results.

### 3.2. PLA Method

#### 3.2.1. PLA Model

**Figure 9:**
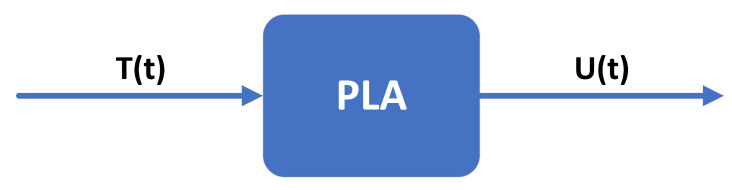
The PLA model.

The PLA method uses the MEMS accelerometer’s temperature and TDO as the input and output of the PLA model, respectively. The model can be described as follows:

(18) $$Y\left(t\right)=U\left(t\right)=F_{L}\left[X\left(t\right)\right]=F_{L}\left[T\left(t\right)\right]$$

where X(t) and Y(t) are the input and output of the PLA model at time *t*, respectively. U(t) and T(t) are the TDO and temperature output of the MEMS accelerometer at time *t*, respectively. $F_{L}\left[\cdot \right]$ is the target function to be trained. The framework of the PLA model is shown in Figure 9.

#### 3.2.2. PLA Algorithm

The MEMS accelerometer continuously outputs the TDO and the measured internal temperature value during the experiment. The measured internal temperature increases as the temperature rises in the temperature-controlled oven. Consider each temperature (T(t)) and its corresponding MEMS accelerometer’s TDO (U(t)) as a whole ([T(t),U(t)]). Divide the data set ([T,U]) evenly into intervals over the entire temperature range according to the temperature. In this paper, we divide the data set into ten intervals, that is $\left[T_{1}\left(t\right),U_{1}\left(t\right)\right],\ldots ,\left[T_{10}\left(t\right),U_{10}t\right)]$. It is fitted separately according to Formula (19) in each interval.

(19) Y(t)=U(t)=kX(t)+b=kT(t)+b

where X(t) and Y(t) are the input and output of the formula at time *t*, respectively. U(t) and T(t) are the TDO and temperature output of the MEMS accelerometer at time *t*, respectively., and *k* and *b*, respectively, denote the slope and intercept of the linear function.

Ten sets of *k* and *b* and nine temperature split points will be calculated using the PLA algorithm to fit the training data. The temperature values in the validation data set are first determined by which temperature range they belong to according to the temperature split points. The predictions are then calculated by combining the corresponding PLA parameters.

## 4. Results and Analysis

This paper used a calibrated GNC Module to experiment, during which the data collecting process lasted at least 2 hours, and more than 1,440,000 data were obtained. We collected two sets of TDO of a MEMS accelerometer under the same condition, one for training the model proposed in this paper, named training data, and the other for validating the performance of the previously obtained model, named validation data. In this way, we verified the compensation effect of the proposed methods in this paper at a heating rate of 1 °C/min. Therefore, the two data sets have similar characteristics with at least half an hour between experiments. The experimental scheme is described in Section 2.2. The training and validation process on the proposed model are both based on a CPU. All the simulations and analyses in this paper were carried out in Matlab.

**Figure 10:**
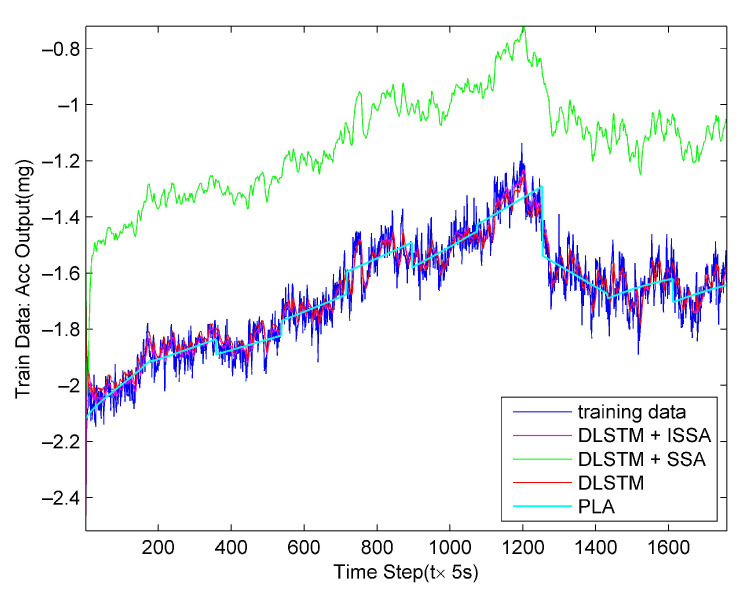
Simulation results on training data by the four methods.

We performed 5 s smoothing on the training data in the training process to remove noise caused by data fluctuations. The hidden layers’ node numbers were both ten. The DLSTM’s maximum number of iterations was set to 500. The preset cycles of SSA and ISSA were both 200. The learning rate was 0.02. The initial population of DLSTM had ten individuals. Figure 10 shows the simulation results of the training data. From Figure 10, we can see that all four methods simulate the training data well, in which the bias of the DLSTM + SSA method is the largest. At this time, we obtained three set global optimal parameters of the DLSTM by the three methods and a set global optimal parameters of the PLA.

**Figure 11:**
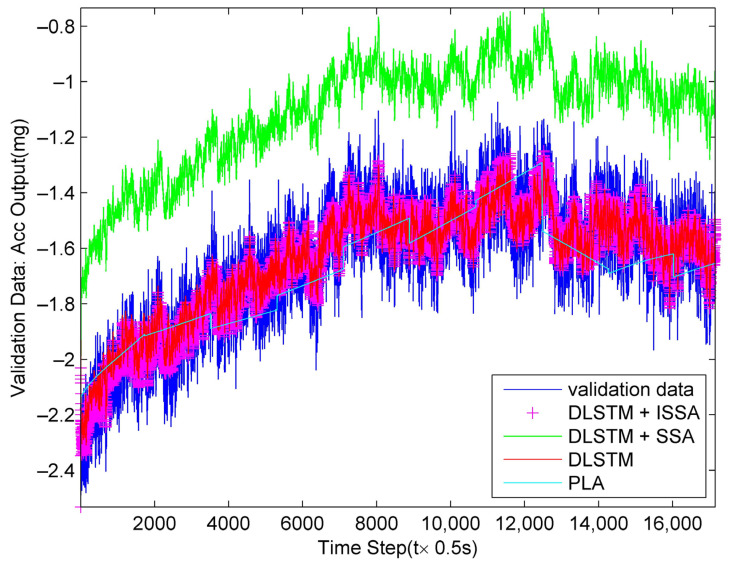
Fitting results on validation data by the four methods.

**Figure 12:**
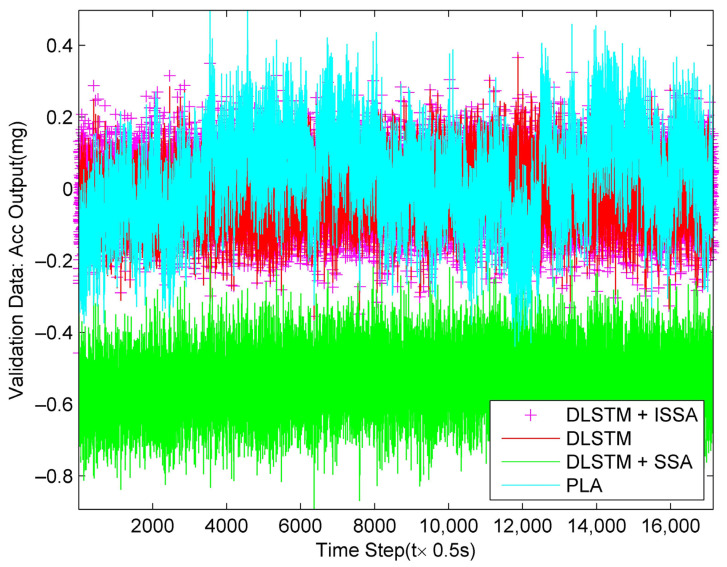
Compensation results on validation data by the four methods.

Then, we apply the four models newly obtained from training data on validation data to verify their effectiveness. We perforedm 0.5 s smoothing on the validation data to ensure its diversity. Figure 11 shows the fitting results, and Figure 12 shows the compensation results. From Figure 11 and Figure 12, all of the methods work well except for the bias of the DLSTM + SSA method.

**Figure 13:**
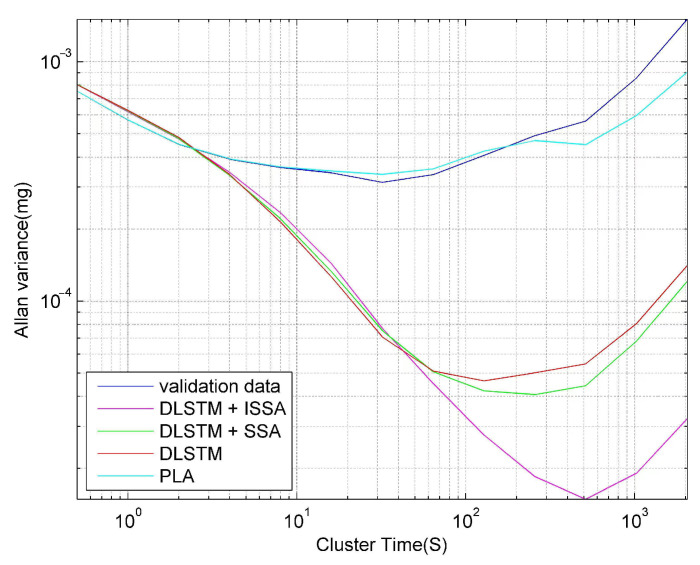
Allan variance analysis results after compensation of validation data by the four methods.

Furthermore, we introduce Allan variance to analyze the compensation results quantitatively. Allan variance is a standard and popular method for analyzing the performance of inertial sensors [26,43,44]. Figure 13 shows the compensation results of Allan variance analysis by the four methods, and Table 1 shows the corresponding quantitative results. Here, we mainly focus on the three Allan coefficients: bias instability (*B*), rate random walk (*K*), and rate ramp (*R*).

Figure 13 shows that the DLSTM + ISSA method shows the best compensation performance, further confirmed by Table 1. Table 1 shows that after compensation by the four methods, the three Allen variance coefficients increased by an average of 89.36%, 90.85%, 96.68%, and 17.94%, respectively. The results also prove the effectiveness of the proposed method.

## 5. Conclusions

This paper investigates a MEMS accelerometer’s temperature drift compensation methods based on DLSTM, DLSTM + SSA, and DLSTM + ISSA. First, we proposed a real-time compensation model, a recursive method. Then, DLSTM was established to improve the accuracy of the temperature drift compensation model. DLSTM + SSA and DLSTM + ISSA were used to optimize the parameters of DLSTM to improve its precision, where ISSA was an optimization algorithm for SSA. Moreover, the linear approximation model and PLA method were also used to compare with the proposed methods in this paper. Finally, Allan variance analysis was used to quantify the compensation results, showing that all three methods had excellent performance of which DLSTM + ISSA was the best. The PLA method has little compensation effect in this application. The results of Allan variance analysis showed *B* from $5.43\times 10^{-4}$ to $2.77\times 10^{-5}$, *K* from $4.33\times 10^{-5}$ to $1.14\times 10^{-6}$, and *R* from $1.18\times 10^{-6}$ to $2.63\times 10^{-8}$ after compensation by DLSTM + ISSA. These three coefficients have an average improvement of 96.68%.

## Figures and Tables

**Table 1:** Allan variance analysis for different compensation methods.

	Validation Data	DLSTM	Improvement (%)	DLSTM + SSA	Improvement (%)	DLSTM + ISSA	Improvement (%)	PLA	Improvement (%)
*B* (mg)	$5.43\times 10^{-4}$	$6.97\times 10^{-5}$	87.16	$6.33\times 10^{-5}$	88.35	$2.77\times 10^{-5}$	94.89	$5.25\times 10^{-4}$	3.29
$K\;({\mathrm{mg}/s}^{\frac{1}{2}})$	$4.33\times 10^{-5}$	$4.19\times 10^{-6}$	90.33	$3.39\times 10^{-6}$	92.16	$1.14\times 10^{-6}$	97.37	$3.45\times 10^{-5}$	20.23
R(mg/s)	$1.18\times 10^{-6}$	$1.11\times 10^{-7}$	90.59	$9.40\times 10^{-8}$	92.05	$2.63\times 10^{-8}$	97.77	$8.24\times 10^{-7}$	30.31
Aver (%)			89.36		90.85		96.68		17.94

## Data Availability

Not applicable.

## Abbreviations

The following abbreviations are used in this manuscript:

MEMS	Micro-electro-mechanical system
DLSTM	Deep Long Short-term Memory Recurrent Neural Network
SSA	Sparrow Search Algorithm
ISSA	Improved Sparrow Search Algorithm
TDO	temperature drift output
IMU	Inertial Measurement Unit
BP NN	back propagation neural network
AGA	adaptive genetic algorithm
GA	genetic algorithm
PSO	particle swarm optimization
RBF NN	radial basis function neural networks
GNC	guidance navigation and control
PLA	piecewise linear approximation
TDE	temperature drift error
KF	Kalman filter
AFS	artificial fish swarm
LF	Lévy flight
